# Improving Antibiotic Stewardship Through a Retrospective Study of the Diagnostic Value of Nasal Methicillin-Resistant Staphylococcus aureus Polymerase Chain Reaction for Pneumonia in Intensive Care Unit Settings

**DOI:** 10.7759/cureus.116050

**Published:** 2026-09-10

**Authors:** Jasmin K Bhullar

**Affiliations:** 1 Department of Internal Medicine, University of North Carolina Health Southeastern, Lumberton, USA

**Keywords:** antibiotic stewardship, medical intensive care unit (micu), mrsa pneumonia, pneumonia, polymerase chain reaction

## Abstract

Methicillin-resistant *Staphylococcus aureus* (MRSA) is a major cause of pneumonia in intensive care unit settings. Nasal swabs with subsequent polymerase chain reaction (PCR) are commonly performed in hospitals and intensive care units to screen for MRSA nasal colonization. A retrospective data analysis of patients presenting to the intensive care unit at the University of North Carolina Health Southeastern between January 1, 2024, and December 31, 2024, was performed. The goal was to evaluate the correlation between MRSA PCR results from nasal swabs and the presence of MRSA in lower respiratory cultures. The sensitivity of MRSA nasal swabs in the intensive care unit for the growth of MRSA in lower respiratory cultures was 90% (18/20). The positive predictive value of MRSA nasal swabs for the growth of MRSA in lower respiratory cultures was 60% (18/30). These retrospective study data indicate that an MRSA nasal swab may have good clinical utility. A negative MRSA nasal swab may substantially lower the likelihood of MRSA in a lower respiratory culture. These findings also indicate that a positive MRSA nasal swab alone may not mandate anti-MRSA therapy. However, large-scale studies across diverse intensive care settings are necessary to confirm these findings.

## Introduction

Methicillin-resistant *Staphylococcus aureus* (MRSA) is a major cause of severe infections, such as pneumonia, among hospitalized patients [1]. This particularly applies to intensive care units (ICUs). Early detection and initiation of treatment for MRSA pneumonia are crucial for patient outcomes.

The prevalence of MRSA infections and multidrug-resistant organisms has been rising [2]. This underscores the importance of reliable diagnostic methods to guide antibiotic therapy. Nasal swabs with polymerase chain reaction (PCR) testing for MRSA are commonly performed in hospitalized patients and ICU patients to screen for MRSA nasal colonization. Their utility in the local ICU remains unclear.

Previous research suggests that PCR testing for MRSA in the nasopharyngeal cavity has a high negative predictive value (NPV) for ruling out MRSA pneumonia [3]. Previous literature also suggests that a negative nasal MRSA swab result has a high negative predictive value for ruling out the growth of MRSA in lower respiratory cultures [4]. However, research data specific to ICU patients are limited. Often, these studies do not include general ICU patients but are restricted to specific ICU populations. A retrospective cohort study that was restricted to 136 burn-injured ICU patients showed a sensitivity of 88%, a positive predictive value of 35.4%, and a negative predictive value of 99.2% for pneumonia caused by infection with MRSA [5]. Another smaller single-center retrospective study in a surgical ICU correlated MRSA nasal PCR testing with MRSA pneumonia. It was restricted to surgical patients. The study demonstrated a low sensitivity of 29%, a positive predictive value of 25%, and a negative predictive value of 90.6%. These studies show that there is wide variability in the reported values regarding the utility of MRSA nasal PCR testing in ICU settings [6]. This study aims to assess the correlation between nasal MRSA PCR results and MRSA pneumonia in ICU patients at the University of North Carolina Health Southeastern. The aim is to evaluate these findings within the local ICU population to inform clinical practice at this institution.

## Materials and methods

Study design and objective

The present study is a retrospective cross-sectional study. The goal was to evaluate the diagnostic concordance between MRSA PCR results from nasal swabs and the presence of MRSA in lower respiratory cultures. The broader aim was to support a quality improvement initiative evaluating the potential utility of MRSA nasal swab PCR testing in the local ICU to guide clinical management. This would support improved antimicrobial stewardship by informing more targeted antibiotic use.

Study population

This study included adult patients admitted to the ICU at the University of North Carolina Health Southeastern who underwent nasal swab collection for MRSA PCR testing and lower respiratory culture between January 1, 2024, and December 31, 2024. All of the following inclusion criteria had to be met: ICU admission during the hospitalization, age ≥18 years, and collection of both MRSA PCR samples and lower respiratory cultures during the same hospitalization. Exclusion criteria included patients under 18 years of age.

Ethical considerations

The Institutional Review Board (IRB) of the University of North Carolina Health Southeastern approved this study (25.382).

Study variables and outcome measures

The collected data included information on the times of arrival at and discharge from the intensive care unit at the University of North Carolina Health Southeastern, results of PCR testing for MRSA from nasal swabs, and results of lower respiratory cultures. The PCR results were obtained using the Food and Drug Administration (FDA)-cleared Cepheid Xpert MRSA assay, a qualitative in vitro diagnostic test intended for the detection of MRSA deoxyribonucleic acid (DNA) directly from nasal swabs. The test utilizes automated real-time PCR for the amplification of MRSA-specific DNA targets and fluorogenic target-specific hybridization probes for the real-time detection of the amplified DNA. The outcomes included the number of patients with abnormal respiratory cultures, the number of patients with growth of MRSA in their lower respiratory cultures, the number of patients with absence of growth of MRSA in their lower respiratory cultures, the number of patients testing positive for MRSA on nasal swab PCR testing, the number of patients testing negative for MRSA on nasal swab PCR testing, the sensitivity of MRSA nasal swabs in the ICU for growth of MRSA in the lower respiratory culture, and the positive predictive value of a positive MRSA nasal swab for the growth of MRSA in the lower respiratory culture.

Statistical analysis

The data were analyzed using descriptive statistics with the calculation of percentages, sensitivities, and positive predictive values.

## Results

Seventy-seven patients had an abnormal respiratory culture among all the patients who were admitted to the intensive care unit at the University of North Carolina Health Southeastern during 2024. Forty-four of them had growth of species other than Candida or MRSA. Seventeen had growth of Candida species in their lower respiratory cultures. Twenty-one of the 77 patients had growth of MRSA in their lower respiratory cultures. These categories are not mutually exclusive because some patients with abnormal respiratory cultures had polymicrobial lower respiratory cultures (Table 1). 

**Table 1:** Overview of abnormal respiratory culture results MRSA: methicillin-resistant *Staphylococcus aureus*.

Result of lower respiratory culture	Number of patients with this result
Abnormal respiratory culture	77
Growth of species other than *Candida *or MRSA	44
Growth of *Candida *species	17
Growth of MRSA	21

Overall, 67 patients tested positive for MRSA based on nasal MRSA PCR testing among the patients who were admitted to the ICU during 2024. Of those 67 patients, 30 underwent additional collection of lower respiratory cultures during the same hospitalization. Among those 30 patients, 18 tested positive for MRSA in lower respiratory cultures, and 12 tested negative for MRSA in these cultures (Table 2).

**Table 2:** Overview of results among patients who were tested positive for MRSA on nasal swab PCR testing MRSA: methicillin-resistant *Staphylococcus aureus*, PCR: polymerase chain reaction.

Result	Number of patients with this result
Tested positive for MRSA on nasal swab PCR testing	67
Tested positive for MRSA on nasal swab PCR testing and positive for MRSA in lower respiratory cultures	18
Tested positive for MRSA on nasal swab PCR testing and negative for MRSA in lower respiratory cultures	12

Twenty of the 21 ICU patients at the University of North Carolina who had growth of MRSA in their lower respiratory cultures had also undergone screening for MRSA with nasal swab testing during the same hospital stay. Eighteen of those 20 patients had a positive result on nasal swab MRSA PCR testing, and two had a negative result. One patient did not undergo nasal PCR testing for MRSA and was therefore excluded from the statistical analysis for the calculation of sensitivity (Table 3).

**Table 3:** Overview of MRSA nasal swab PCR testing results among all intensive care unit patients with growth of MRSA in their lower respiratory cultures MRSA: methicillin-resistant *Staphylococcus aureus*, PCR: polymerase chain reaction.

Result	Number of patients with this result
Tested positive for MRSA on lower respiratory cultures	21
Tested positive for MRSA on lower respiratory cultures and positive for MRSA on nasal swab PCR testing	18
Tested positive for MRSA on lower respiratory cultures and negative for MRSA on nasal swab PCR testing	2
Tested positive for MRSA on lower respiratory cultures and not tested for MRSA with nasal swab PCR testing	1

The sensitivity of an MRSA nasal swab in the intensive care unit at the University of North Carolina Health Southeastern for the growth of MRSA in lower respiratory cultures was 90% (18/20).

The positive predictive value of a positive MRSA nasal swab for the growth of MRSA in lower respiratory cultures was 60% (18/30).

These findings suggest that a negative MRSA nasal swab at the University of North Carolina Health Southeastern substantially decreases but does not completely eliminate the likelihood of MRSA growth in lower respiratory cultures.

## Discussion

The present retrospective study shows that MRSA nasal swab PCR testing has a high sensitivity of 90% for the growth of MRSA in lower respiratory cultures and a modest positive predictive value of 60%. Overall, these findings are consistent with prior evidence from non-ICU settings. A meta-analysis of the clinical utility of MRSA nasal screening to rule out MRSA pneumonia included 21 studies, comprising 5163 patients. This meta-analysis did not focus specifically on ICU patients. The pooled sensitivity of the MRSA nares screen for MRSA pneumonia in this meta-analysis was 70.9%, and the calculated positive predictive value was 44.8% [7]. The values for sensitivity and positive predictive value in the present study are higher than those in this meta-analysis. This is also likely because the present study correlates the results of MRSA nasal testing with testing for MRSA in lower respiratory cultures instead of MRSA pneumonia. Lower respiratory cultures may capture both airway colonization and true infection, which may result in higher sensitivity than studies applying strict clinical and radiographic criteria for the diagnosis of MRSA pneumonia. Data from ICU settings on the utility of MRSA nasal screening are limited. One retrospective study of 210 patients in the intensive and intermediate care units correlated nasal swab MRSA PCR testing with isolation of MRSA from respiratory cultures. The sensitivity in that study was 90.5%, the positive predictive value was 34.5%, and the negative predictive value was 98.6% [8]. These findings are similar to those from the present study. Another small retrospective study demonstrated a low sensitivity of 29%, a low positive predictive value of 25%, and a high negative predictive value of 90.6%. This small retrospective study correlated the data from MRSA nasal PCR testing with MRSA pneumonia among surgical ICU patients [6]. In contrast, data from the local institution were not restricted to surgical or medical ICU patients. They contained data from both patient populations. Our recent data from the ICU setting at the University of North Carolina Health Southeastern are overall consistent with prior research from non-ICU and ICU settings. However, the values for sensitivity and predictive value in the surgical ICU seem to be lower than those in the present study. This may be due to a different clinical risk profile of surgical patients specifically [6].

Overall, the results indicate that nasal MRSA PCR testing has good utility as a diagnostic tool to facilitate antibiotic stewardship. It may improve antibiotic treatment decisions in ICU settings. A large retrospective cohort study analyzing 561,325 clinical cultures has established that MRSA nares screening had high NPVs regarding various MRSA infections, which are not restricted to the respiratory tract. The negative predictive values of MRSA screening regarding subsequent clinical cultures for MRSA were >90% for bloodstream infections, intra-abdominal cultures, respiratory cultures, wound cultures, and urinary cultures [9]. High sensitivities in combination with high negative predictive values of MRSA nasal swabs could potentially reduce the prescription of unnecessary broad-spectrum antibiotic therapy in ICU settings. A negative swab may support avoidance or even narrowing of already started empiric MRSA coverage in clinically stable patients with a low-to-moderate pretest probability of MRSA pneumonia. This could promote antibiotic stewardship. It would also be beneficial in terms of reducing the risk of antimicrobial resistance [10]. Larger studies are needed to confirm this finding.

If these findings are confirmed in large-scale studies, this would support using MRSA nasal swabs as additional data points to justify early discontinuation of unnecessary antibiotics. This would reduce unnecessary prescriptions of vancomycin and linezolid and reduce side effects such as nephrotoxicity or hematologic toxicity [11,12].

However, data also suggest that the positive predictive value of a positive nasal swab is only modest. This indicates that a positive MRSA nasal swab alone should not mandate antibiotic coverage directed against MRSA (e.g., vancomycin or linezolid).

This research supports the use of nasal MRSA PCR as a routine screening tool for hospitalized patients in the intensive care unit setting. However, caution is advised. Anti-MRSA coverage for pneumonia should be guided by the severity of the patient's clinical condition, risk factors for MRSA such as prior MRSA infection or colonization, recent hospitalizations, recent use of intravenous antibiotics, comorbidities, and radiographic features.

Larger-scale studies could inform antibiotic stewardship practice at the University of North Carolina Health Southeastern and potentially also improve antibiotic prescription practices in similar ICU settings.

Limitations

This study has several limitations. First, it was a retrospective cross-sectional study, and observed associations cannot be interpreted in a causal or temporal manner. Second, the data were collected at a single center over a one-year period and may not be fully applicable to other intensive care units. Third, lower respiratory cultures were not collected from all patients who underwent nasal MRSA PCR testing. This may have introduced selection bias. Fourth, the results from MRSA nasal swab PCR testing were only correlated with the results for MRSA in lower respiratory cultures and not directly with the ICD coding for MRSA pneumonia itself. The presence of MRSA in a lower respiratory culture does not necessarily establish MRSA pneumonia. The present study also did not report negative predictive values or specificities for the correlation between MRSA nasal swab PCR testing and MRSA testing in lower respiratory cultures. Finally, the sample sizes were relatively small. This limits the precision of the estimated sensitivity and positive predictive value. The findings of this retrospective study should be interpreted within the context of these limitations. Large-scale studies would help validate and extend these results across different ICU settings.

## Conclusions

The data from this retrospective analysis of the intensive care unit setting over a single year at the University of North Carolina Health Southeastern suggest that MRSA nasal swabs may be particularly useful in reducing the post-test probability of MRSA pneumonia if they are negative in patients with a low-to-moderate pretest probability. However, the data suggest that anti-MRSA therapy should not be based on a positive MRSA nasal swab alone, given the modest positive predictive value.

These retrospective study data indicate that MRSA nasal swabs may have good clinical utility. However, large-scale studies in other intensive care settings are necessary to confirm these findings. If these findings are confirmed in large-scale studies, this would support early narrowing of the antibiotic regimen in patients with a negative MRSA nasal swab who are clinically stable.
